# Efficient Multi-Fidelity Fluid–Structure Interaction Modeling for Pulsatile Blood Flow in Deformable Biological Tissues

**DOI:** 10.1007/s10439-025-03966-x

**Published:** 2026-03-11

**Authors:** Chang Min Lee, Youngjae Choi, Kiwon Lee, Mihyun Lee, Seung-Hoon Kim, Yong-Soon Yoon, Hyun Jin Kim

**Affiliations:** 1 Department of Mechanical Engineering, Korea Advanced Institute of Science and Technology, Daejeon, Republic of Korea; 2 Ceragem Clinical Inc. Ceragem Clinical Research Institute, Gwacheon, Republic of Korea; 3 Department of Rehabilitation Medicine, Presbyterian Medical Center, Jeonju, Republic of Korea

**Keywords:** Blood flow, Fluid structure interaction, Reduced order model, Compression therapy

## Abstract

****Purpose**:**

Simulating tissue deformation and flow alterations induced by external compression typically requires fluid–structure interaction (FSI) analysis, which is computationally demanding. This study presents a multi-fidelity FSI framework that efficiently captures tissue mechanics and hemodynamic responses to dynamic external pressure and demonstrates its applicability to compression therapy.

****Methods**:**

We developed an FSI model that couples a one-dimensional deformable blood flow formulation with the three-dimensional (3D) Cauchy equation of motion. Model performance was evaluated by comparing the multi-fidelity and full FSI solutions in simplified cylindrical and subject-specific geometries. As a practical demonstration, the framework was applied to simulate a full-cycle pulsatile intermittent pneumatic compression (IPC) operation.

****Results**:**

The model efficiently reproduced tissue deformation and hemodynamic changes under external compression, yielding <1% flow-rate error in both geometries and <2% pressure error in the simplified geometry for most of the cycle, with good agreement in the subject-specific geometry. Computational cost was reduced by a factor of 9 in the cylindrical geometry and 46 in the subject-specific geometry relative to full 3D FSI. In the IPC application, the model captured dynamic behavior over an extended temporal scale, completing a full cycle in 457 s for the simplified geometry and 42.2 min for the subject-specific geometry.

****Conclusion**:**

This multi-fidelity FSI framework enables efficient and accurate simulation of tissue deformation and hemodynamic responses under external pressure, providing a tractable platform for large-scale parametric and optimization studies. Its application to IPC highlights potential to enhance therapeutic device design and support broader applications in biomedical modeling and medical device development.

**Supplementary Information:**

The online version contains supplementary material available at https://doi.org/10.1007/s10439-025-03966-x.

## Introduction

Understanding the deformation of biological tissues and changes in fluid flow in response to external compression has long been a subject of interest. These phenomena are ubiquitous in diverse clinical contexts, including blood pressure measurement, compression therapy, and respiration. Various computational approaches have been employed to comprehensively understand the mechanisms and interactions of biological systems. These simulations necessitate fluid–structure interaction (FSI) methods as the structural deformation and fluid dynamics are closely coupled, influencing each other.

Among FSI methods applied to biological systems, the arbitrary Lagrangian–Eulerian approach [1–3], immersed boundary methods [4], and other related techniques have been widely employed [5–7]. However, their practical use is often constrained by high computational demands. To address this limitation, considerable research has focused on multi-fidelity reduced-order models, which enable the analysis of complex systems within feasible computational timescales.

For pulsatile blood flow simulations, structural domains are frequently simplified to reduce computational cost. For instance, vascular walls have been represented using shell elements [8, 9] or further reduced to thin-membrane models, where wall deformation is approximated via pressure–area relationships [10, 11]. Similarly, the complex motion of localized structures such as heart valve leaflets has been captured using low-degree-of-freedom models [12, 13]. These approaches are well suited for deformable vascular tissues but may be less applicable to thick, structurally complex biological materials. Conversely, when solid deformation is modeled in greater detail, blood flow and pressure fields are often approximated using zero- or one-dimensional (1D) reduced-order models, which are then coupled to three-dimensional (3D) structural representations such as electromechanical models of the heart [14, 15] or poroelastic models of the lower limb [16, 17].

When biological tissues are subjected to external compression, the resulting deformation can be substantial enough to alter blood flow. In such cases, multi-fidelity reduced-order models must support two-way coupling between the fluid and structural domains. Appropriate boundary conditions are also essential, as local hemodynamics are strongly influenced by parts of the biological system outside the modeled computational domain. Several studies have used such models to investigate tissue deformation and flow alterations under internal and external loading.

For instance, compression therapy has been examined using different forms of multi-fidelity reduced-order models. Some studies have focused primarily on solid mechanics, computing tissue deformation and stress while applying simplified representations of blood flow [18, 19], thereby oversimplifying fluid–structure interaction. Others have modeled blood flow using fixed geometries representing compressed and uncompressed states [20, 21], neglecting the dynamic evolution of tissue deformation. Alternatively, one-dimensional FSI models based on pressure–area relationships have been applied [22, 23]. These approaches are computationally efficient and suitable for long-time simulations but are limited in their ability to realistically capture complex three-dimensional tissue deformation, and they require careful specification of boundary conditions.

To address these limitations, a one-dimensional separated-flow model for collapsible tubes [24, 25] has been coupled with three-dimensional structural analysis to simulate tissue deformation and airflow in the vocal folds [26, 27]. This framework enabled efficient computation while accounting for three-dimensional tissue deformations. Its application, however, was restricted to a simplified geometry of a single conduit undergoing small compression-induced deformations, primarily within the context of vocal fold dynamics. The boundary conditions employed were correspondingly simple, reflecting the single-conduit domain. To date, this approach has not been extended to more complex biological flow systems—such as blood flow in extensive vascular networks—nor has it been applied to scenarios involving large-scale tissue deformation and flow alterations under substantial external compression.

Alternatively, ventricular-induced compression of coronary microvessels and the resulting flow alterations have been investigated using coupled three-dimensional Darcy flow and electromechanics equations [28, 29]. These studies assessed the effects of cardiac contraction on microvascular perfusion in a lumped representation and evaluated its influence on three-dimensional epicardial coronary arteries. Although vessel deformation due to cardiac contraction was not modeled explicitly, a multiscale framework was employed to indirectly account for deformation effects.

For a multi-fidelity reduced-order model to be practically applicable to dynamic flow problems under external compression, it must achieve sufficient accuracy to capture tissue deformation and hemodynamic alterations while maintaining computational efficiency. In this study, we introduce a multi-fidelity FSI model that explicitly couples a one-dimensional deformable blood flow formulation with the three-dimensional Cauchy equation of motion to efficiently and accurately simulate the anatomically dependent deformation of heterogeneous, anisotropic biological tissues and the resulting fluid flow changes under external pressure. The proposed framework is compared with full 3D FSI simulations in both simplified cylindrical and subject-specific geometries to evaluate its accuracy and computational efficiency.

The model is further applied to analyze intermittent pneumatic compression (IPC). To represent venous inflow more realistically, a lumped-parameter model of the distal venous system is implemented as the inlet boundary condition. Using this framework, we investigate tissue deformation and blood flow alterations during IPC operation and elucidate the underlying mechanisms of device action. Results demonstrate that the proposed model captures tissue deformation and flow dynamics under external compression with high efficiency, providing a practical tool for parameter studies, medical device development, and the design of patient-specific therapeutic strategies.

## Materials and Methods

### Multi-fidelity Fluid–Structure Interaction Model

The one-dimensional deformable blood flow equations and the three-dimensional Cauchy equation of motion are explicitly coupled to calculate tissue deformations and changes in blood flow and pressure due to external pressure.

**Fig. 1:**
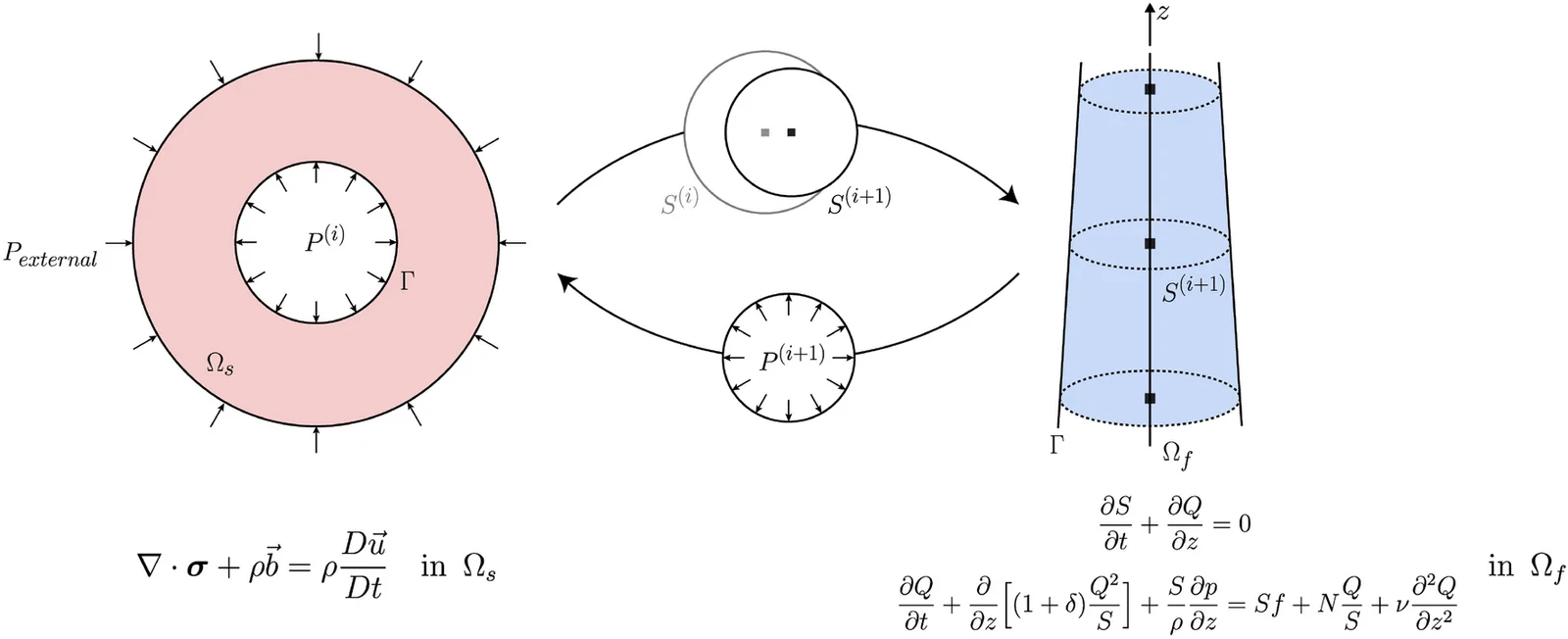
Schematic of the multi-fidelity fluid–structure interaction model. $\Omega _s$ and $\Omega _f$ represent the solid and fluid domains, respectively, and Γ denotes the interface between these domains. The structural analysis and blood flow simulation are governed by the Cauchy momentum equation and the 1D deformable blood flow equation, respectively. $P_{external}$ denotes external pressure, while *p* is the fluid pressure exerted on the interface. *S* indicates the cross-sectional area at each point within the 1D fluid domain. The cross-sectional area *S* and pressure *p* are iteratively updated based on the calculated deformation and fluid pressure.

For the fluid domain $\Omega _f$, assuming incompressibility, impermeability, and axially dominant flow, the mass conservation and momentum balance equations can be expressed as follows [10]:

1 $$\begin{aligned} \frac{\partial S}{\partial t} + \frac{\partial Q}{\partial z}&= 0 \end{aligned}$$

2 $$\begin{aligned} \frac{\partial Q}{\partial t} + \frac{\partial }{\partial z}\Big [(1+\delta ) \frac{Q^2}{S}\Big ] + \frac{S}{\rho }\frac{\partial p}{\partial z}&= Sf + N \frac{Q}{S} + \nu \frac{\partial ^2 Q}{\partial z^2} \end{aligned},$$

where *S* denotes the cross-sectional area, *Q* is the flow rate, *t* represents time, *z* is the axial coordinate, ρ is the density of the fluid, *f* denotes the external force, ν is the kinematic viscosity, and *N* and δ are parameters determined by the velocity profile across the cross section. The fluid domain is represented as a one-dimensional model where the vessels are represented as a collection of consecutive thin slabs. Each vessel cross section is represented with the center location and the radius information, as illustrated in Fig. 1, and is constructed by extracting the centerline from the vascular geometry. The one-dimensional deformable blood flow equations were discretized in space using the finite volume method. The resulting system of equations was solved using sparse LU decomposition and the generalized-α method was adopted for time integration [30].

The three-dimensional Cauchy equation of motion for the solid domain $\Omega _s$ is given by

3 $$\begin{aligned} \nabla \cdot \boldsymbol{\sigma } + \rho \vec {b} = \rho \frac{D\vec {u}}{Dt} \end{aligned},$$

where σ is the Cauchy stress tensor, $\vec {u}$ is the displacement vector, $\vec {b}$ is the body force per unit volume, and ρ is the density of the solid. The solid domain was simulated using svMultiPhysics, a multi-physics finite element method solver [31], and the linear system of equations was solved using the generalized minimal residual (GMRES). Consistent with the fluid domain, the generalized-α method was applied for time integration.

The coupling between fluid and structure was implemented by enforcing continuity of traction and displacement at the interface, with iterative updates to the boundary condition and geometry. With the assumption that the normal stress exerted by blood flow on the vessel wall is significantly higher than the tangential stress values, the contribution of shear stress to traction was neglected and the traction continuity condition was simplified to the following form at the interface Γ:

4 $$\begin{aligned} \boldsymbol{\sigma } \vec {n} \approx -p \vec {n} \end{aligned},$$

where σ is the Cauchy stress tensor of the solid, $\vec {n}$ is the unit normal vector at the interface Γ, and *p* is the fluid pressure. Here, *p* corresponds to the pressure at the closest point in the fluid domain to each node on the interface. The continuity of displacement was satisfied by updating the positions and radii of the points on the centerline. These positions and radii were derived from the interface nodes located on the cross sections corresponding to each centerline point. The position of each point was updated as the average location of its associated interface nodes, and its radius was calculated as the average distance from the updated position to those interface nodes. In cases where a larger time step is adopted for the structural analysis than for the fluid domain, the cross-sectional area is assumed to change linearly in time.

In this study, to address numerical instabilities and enhance convergence, a stabilization technique based on temporal relaxation was employed. For each iteration within each time step, the radius and pressure are updated using a weighted average of the current estimate and the value from the previous time step, as follows:

5 $$\begin{aligned} r_{n}^{(i+1)} =\alpha _r \hat{r}_{n}^{(i+1)} + (1-\alpha _r) r_{n-1} \end{aligned}$$

6 $$\begin{aligned} p_{n}^{(i+1)} = \alpha _p \hat{p}_{n}^{(i+1)} + (1 - \alpha _p) p_{n-1} \end{aligned}.$$

Here, $r_{n}^{(i+1)}$ and $p_{n}^{(i+1)}$ represent the updated values of the radius and pressure at i+1th iteration at time step *n*. $\hat{r}_{n}^{(i+1)}$ and $\hat{p}_{n}^{(i+1)}$ denote the current estimates obtained in this iteration. $r_{n-1}$ and $p_{n-1}$ are the converged values from the previous time step n-1. The parameters $\alpha _r$ and $\alpha _p$ are relaxation factors for the radius and pressure, respectively. The relaxation factors control the weighting between the current estimate and the value from the previous time step, and in this study, they were both set to 0.25. The relaxation factor of 0.25 was heuristically selected based on a parametric study to maintain numerical stability while allowing the geometry to update as much as possible.

**Algorithm 1:**
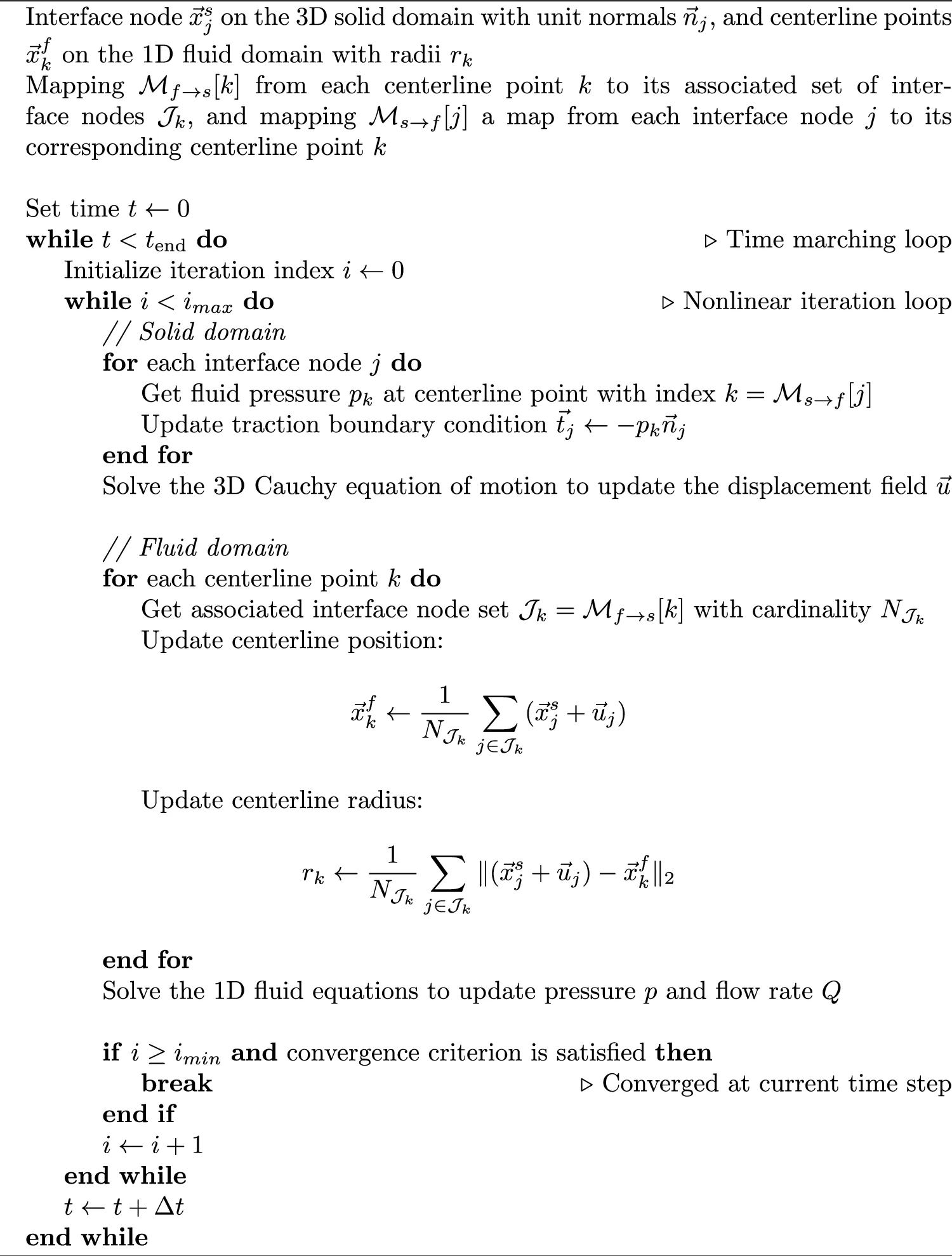
Coupling procedure for the multi-fidelity FSI model

### Lumped-Parameter Network Model of Distal Venous System

**Fig. 2:**
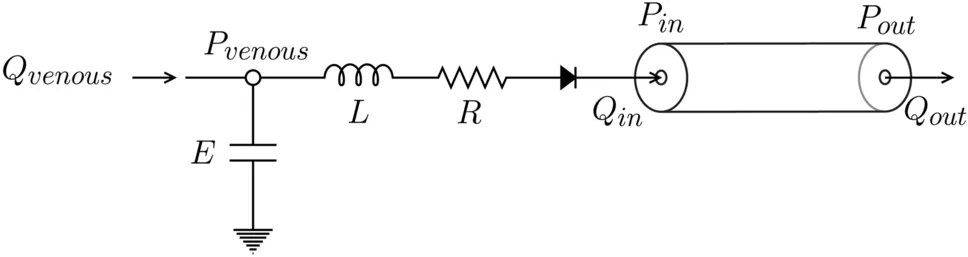
Lumped-parameter network model of the distal venous system. The model represents vessel compliance, inertial effects, and valvular function, and it is assumed that blood flows from the upstream veins into the model. Inlet and outlet flow rates are denoted by $Q_{in}$ and $Q_{out}$, respectively, while inlet and outlet pressures are represented by $P_{in}$ and $P_{out}$. $P_{venous}$ denotes the pressure from accumulated blood within the model, and $Q_{venous}$ represents the inflow into the model. The parameters *E*, *L*, and *R* denote elastance, inductance, and resistance, respectively.

The developed multi-fidelity model was utilized to simulate the operation of the IPC. To obtain reliable simulation results, the appropriate boundary conditions are crucial. A lumped-parameter network (LPN) model was developed to realistically capture the physiology of the distal venous system. As illustrated in Fig. 2, the LPN model was constructed under the assumption that blood flows from the upstream veins into the model, incorporating vessel compliance, inertial effects, and valvular function. This model is governed by the following equations:

7 $$\begin{aligned} Q_{venous} - Q_{in}&= E \frac{dP_{venous}}{dt} \end{aligned}$$

8 $$\begin{aligned} P_{venous} - P_{in}&= RQ_{in} + L \frac{dQ_{in}}{dt} \end{aligned},$$

where $Q_{venous}$ refers to the inflow into the LPN model, $Q_{in}$ is the flow from the LPN model to the inlet of the fluid domain. $P_{in}$ is the pressure at the inlet of the fluid domain, and $P_{venous}$ represents the pressure resulting from blood accumulation within the LPN model. *E*, *L*, and *R* denote elastance, inductance, and resistance, respectively. To ensure unidirectional flow through the venous valve, $Q_{in}$ is set to zero when it becomes negative.

### Simulation Details

For the fluid domain, a parabolic velocity profile was assumed, with $N=-8\pi \nu$ and δ=-1/3. The blood was assumed to have a fluid density of $1.06 \hbox {g/cm}^3$ and the dynamic viscosity of 0.04 poise. For spatial discretization, a point spacing of 0.5 cm was selected based on the grid-independence analysis presented in Supplementary Fig. S1. For temporal discretization, a time step of 0.01 s was chosen to ensure computational feasibility while adequately capturing the system’s transient response. For the solid domain, the material property was defined based on a weighted combination of literature-reported values for fat and muscle tissues, using a 3:1 ratio (fat to muscle) [32, 33]. The resulting elastic modulus was set at E=20580 Pa with Poisson ratio ν=0.47 and density $\rho = 1.0425 \hbox {g/cm}^3$. An isotropic linear elastic model was employed for tissue mechanics to facilitate evaluation of the proposed multi-fidelity coupling strategy and to maintain computational efficiency across full-cycle IPC simulations. Within each time step, variable nonlinear iterations were conducted with a minimum of 2 and a maximum of 5 steps, using a convergence tolerance of 0.001. GMRES was employed with a maximum of 50 iterations, a Krylov subspace dimension of 100, and a tolerance of 0.01.

#### Comparison of the Multi-fidelity Model With Full 3D FSI

The developed multi-fidelity model was compared with full 3D FSI simulations employing the arbitrary Lagrangian–Eulerian formulation for a simple straight cylindrical model and a subject-specific leg model obtained from CT image data. Details on the utilized geometries are provided in the next subsection. Consistent with the structural analysis conducted in the multi-fidelity model, svMultiPhysics was adopted for the full 3D FSI simulation [34]. Computational performance was compared as well. All reported computation times were measured on a computing resource with a 96-core Intel Xeon Platinum 8558 CPU and 256 GB of RAM.

#### Full-Cycle Pulsatile Simulation of Intermittent Pneumatic Compression

Full-cycle pulsatile simulations of intermittent pneumatic compression were conducted using the proposed multi-fidelity model. Details on geometry construction and boundary conditions are as follows.

**Fig. 3:**
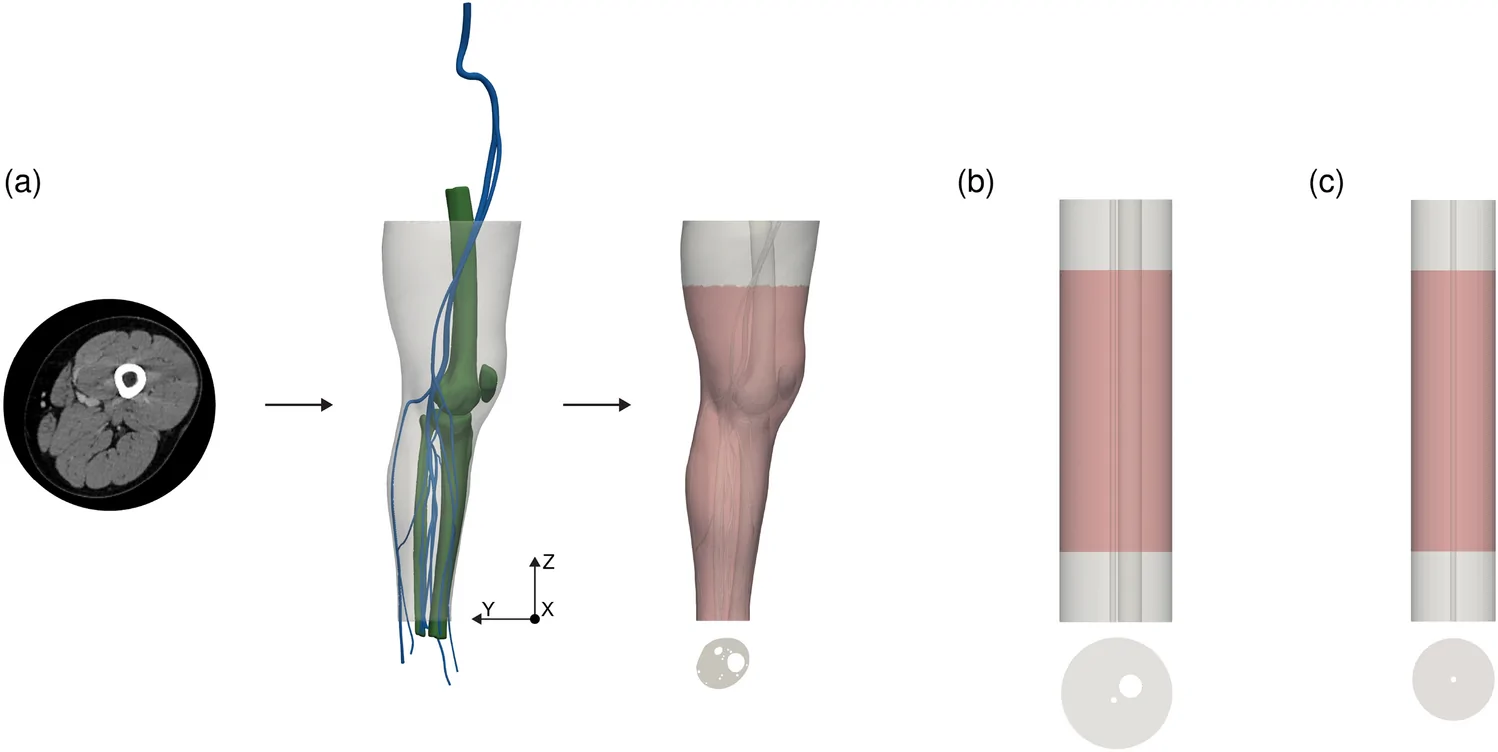
Computational domains representing the lower limb geometry. **a** Subject-specific model. Bone (green), vein (blue), and tissue (white) geometries were extracted from CT images. **b** Simplified single cylindrical model derived from the subject-specific model. **c** Simplified cylindrical model excluding the bone. The regions subjected to external pressure are shown in red.

The geometry of the fluid and solid domains was reconstructed from CT images acquired in the supine position (Fig. 3(a)), with a voxel size of $0.98 \times 0.98 \times 3$ mm. This study was approved by the Institutional Review Board of Jesus Hospital (IRB No. 2023-02-006). The computational domain consists of veins, bones, and tissues. Segmentation was performed on a single leg, from the ankle to the pelvis. The vascular geometry was constructed using SimVascular by lofting the 2D cross sections of the veins [35], and their centerlines were extracted by computing the average position of the points composing each cross section. This configuration naturally establishes a direct mapping between the centerline points and the interface nodes. The bone geometry was extracted using thresholding in 3D Slicer [36]. For the tissue, the geometry was initially acquired by thresholding the entire leg region, from which the vascular and bone geometries were subtracted via a boolean operation in MeshMixer [37].

Given the complexity of the subject-specific geometry, a simplified single cylinder was generated based on the obtained geometry to facilitate the evaluation of the methodology. The simplified cylindrical geometry has a length of 60 cm. The radii of tissue, vein, and bone are 8, 0.4, and 1.7 cm, respectively. While the bone and tissue are centered at the origin, the center of the vein is located 1 cm in the x-direction and 0.4 cm in the y-direction from the origin, as shown in Fig. 3(b).

For comparison with the full 3D FSI simulations, a simplified axisymmetric cylindrical model without bone was utilized in addition to the subject-specific model. This model has a tissue radius of 6 cm, with the center of the vein positioned at the origin, as presented in Fig. 3(c). The tissue radius was adjusted to account for the exclusion of the bone region when constructing the axisymmetric validation model.

For the boundary conditions of both the simplified cylindrical and subject-specific models, a constant central venous pressure (CVP) of 2.54 mmHg was applied at the outlet [38] and the LPN model was utilized for the inlet. Although $Q_{venous}$ is typically dynamic, the total $Q_{venous}$ was set as a constant of 6 mL/s in this study, based on the assumption that the maximum compression pressure has a negligible effect on arterial flow [39]. The parameter values of the LPN model for the inlet of the simplified cylindrical geometry and each inlet of the subject-specific geometry, along with a description of how these values were determined, are provided in Supplementary Fig. S2. For comparison with the full 3D FSI simulations, the specified $Q_{venous}$ was applied as the inlet boundary condition, instead of the LPN model. External pressure was applied to the lateral wall highlighted in red in Fig. 3(a), (b), and (c). To smooth out abrupt transitions, the external pressure profile was processed using a Fourier series expansion. For the inner surface in contact with the fluid, the pressure obtained from solving the governing equations in the fluid domain was applied as a boundary condition. The outlet and the surfaces of the bones were fixed. Lastly, the inlet of the simplified cylindrical model was fixed, while in the subject-specific model, the inlet was constrained only in the z-direction because external pressure was applied starting from the inlet. As the initial condition, prestress was calculated by applying pressure obtained from a steady-state blood flow simulation under the undeformed configuration [40].

## Results

### Comparison of the Multi-fidelity Model with Full 3D FSI

**Fig. 4:**
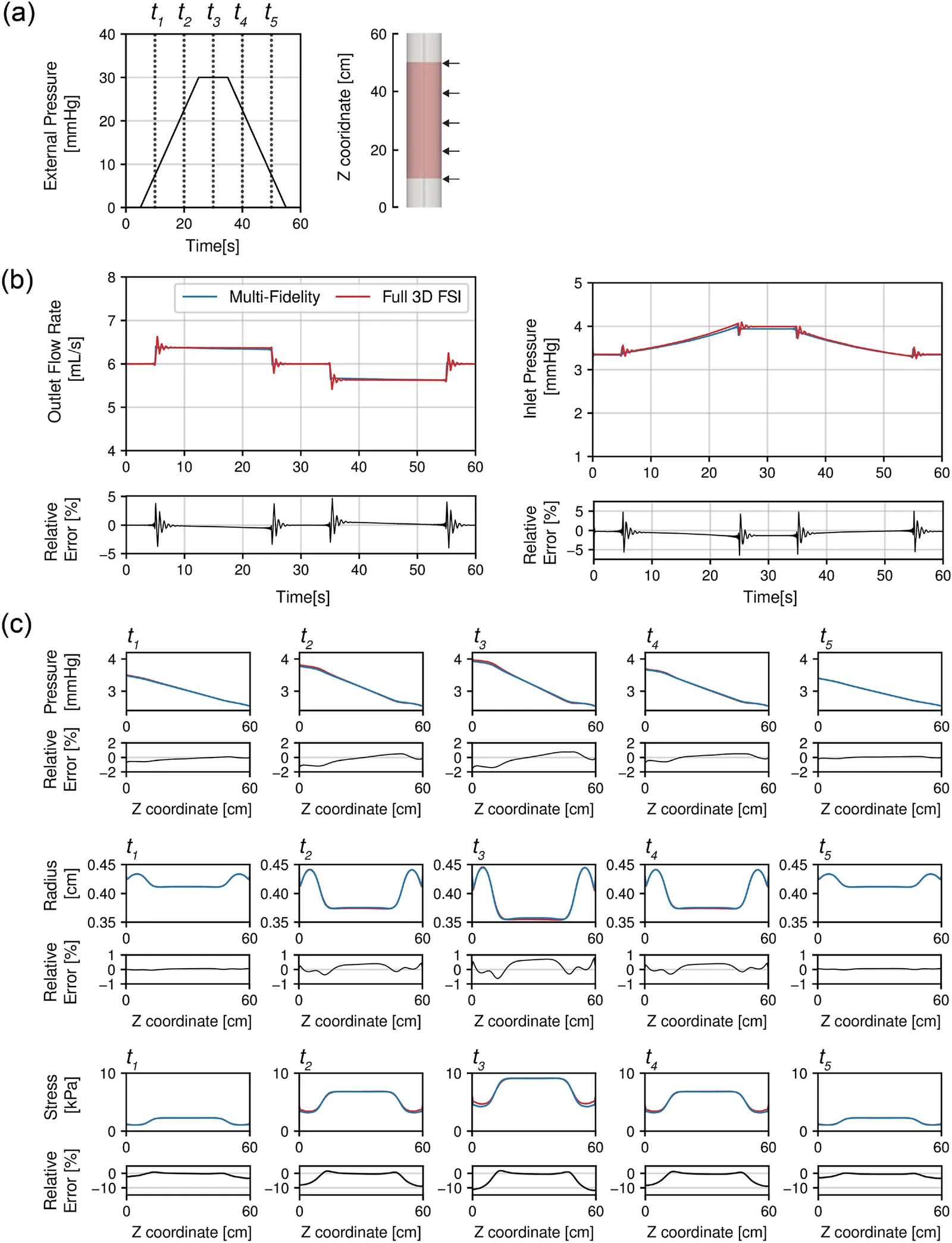
**a** External pressure applied to the simplified cylindrical model. **b** Comparison of the calculated outlet flow rate and inlet pressure with the full 3D FSI simulation, with relative errors shown at the bottom of each panel. **c** Spatial distributions of pressure, radial deformation, and stress magnitude along the centerline at the specified time points ($t_1$-$t_5$), together with the corresponding relative errors. Radial deformation and stress magnitude were averaged across the interface nodes comprising each cross section.

We compared the developed multi-fidelity model with full 3D FSI to assess its accuracy in simulating deformations and blood flow changes caused by external compression with identical simulation configurations. First, the simulations were performed on a simplified cylindrical model by applying the external pressure shown in Fig. 4(a). The volume meshes of the solid and fluid domains were generated with hexahedral elements. The solid mesh consists of 69,212 nodes and 63,360 elements. The fluid mesh for the full 3D FSI simulation contains 21,538 nodes and 18,600 elements. This mesh configuration was selected based on the mesh-independence study presented in Supplementary Fig. S3. The external pressure profile consists of five sequential phases: an initial period with no applied pressure, a gradual ramp-up phase, a constant-pressure plateau, a gradual ramp-down phase, and a final period of zero external pressure. The external pressure profile was selected to reflect the operating mode of IPC devices, with an initial no-pressure phase included to facilitate comparison with the baseline state. Figure 4(b) presents a comparison of the flow rate at the outlet and the pressure at the inlet, respectively. For the flow rate, notable changes are observed at the ends of the phases, where the relative error reaches a maximum of 4.7%. However, the error subsequently decreases and remains within 1% thereafter. Similarly, for pressure, excluding the very beginning of the analysis, the relative error is within a maximum of 5.0%, which occurs at the ends of the phases. The error was progressively reduced for the other phases, remaining within 2% for the remaining period. Figure 4(c) presents the spatial distributions of pressure, radial deformation, and stress magnitude along the centerline at the specified time points. Among these time points, the largest deviations from the full 3D FSI results occur at $t_3$, corresponding to the constant-pressure plateau phase. At this time, the relative error in pressure remains within 2%, and the error in radial deformation is within 1%. For the stress magnitude, the relative error reaches approximately 10% near the fixed inlet and outlet boundaries, whereas it remains within 4% in regions subjected to external pressure and farther from the boundaries. Additional cross-sectional views of the displacement and stress magnitude fields at the selected time points are provided in Supplementary Fig. S4. The full 3D FSI simulation took 8,966 s to complete, whereas the multi-fidelity model required 996 s, corresponding to 11.11% of the computation time of the full 3D FSI.

**Fig. 5:**
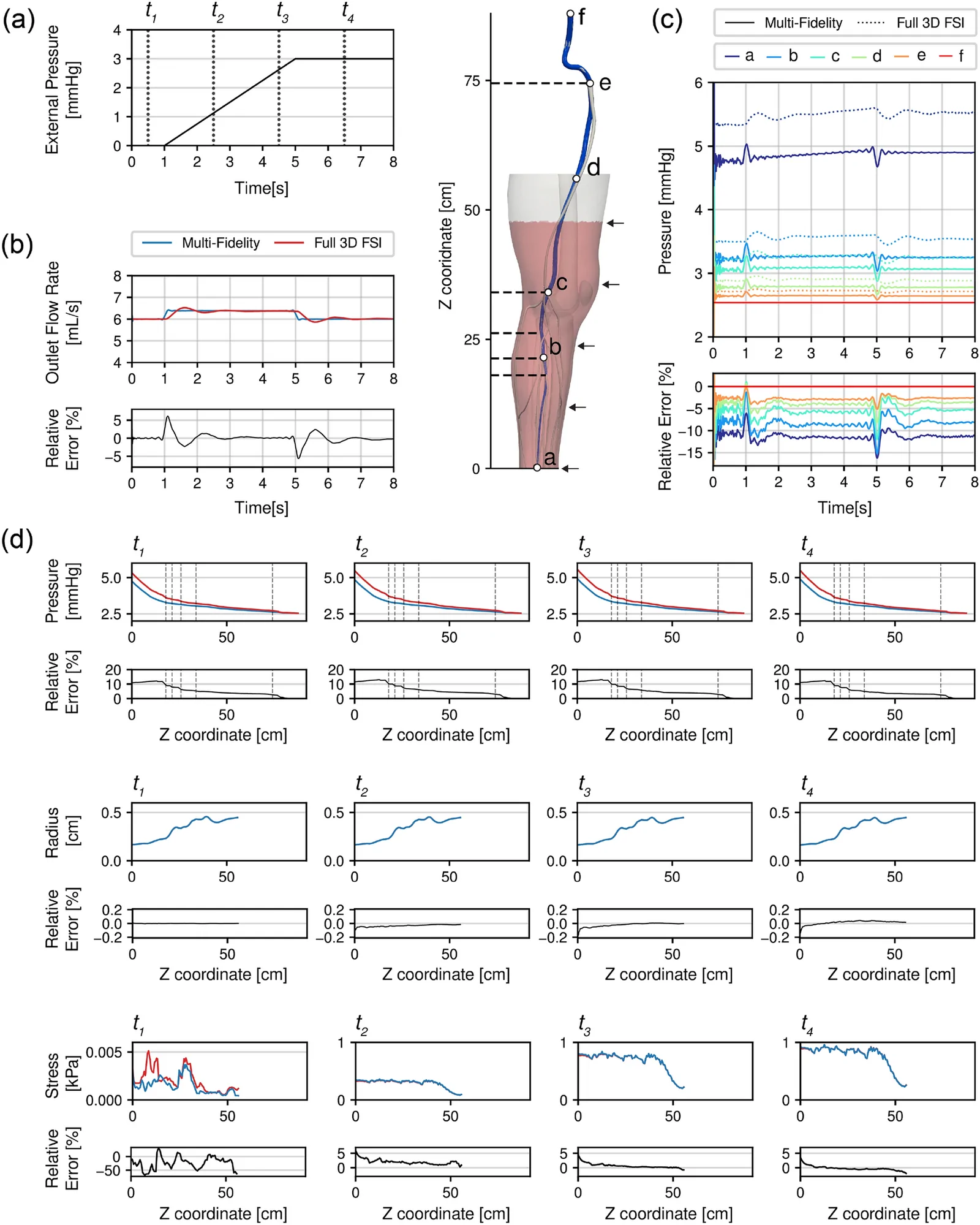
**a** External pressure applied to the patient-specific model. The blue line indicates a path from one of the inlets to the outlet, and dashed lines denote bifurcation locations along this path. **b** Calculated outlet flow rate and **c** pressures at the labeled points (**a–f**), compared with the full 3D FSI simulation results. The labeled points represent key locations along the path, including the inlet (**a**), outlet (**f**), bifurcation points (**b**, **c**, and **e**), and a midpoint (**d**) between points (**c**) and (**e**). **d** Comparison of pressure, radial deformation, and stress magnitude along the blue-marked path with respect to the z-coordinate at the specified time points ($t_1$-$t_4$), together with the corresponding full 3D FSI results. Radial deformation and stress magnitude were averaged across the interface nodes comprising each cross section and are shown only over the solid-domain region. Relative errors are presented at the bottom of each panel.

Additionally, the subject-specific model was employed to compare the simulation results of the multi-fidelity model and the full 3D FSI. The surface mesh was locally refined to adequately resolve anatomical features, yielding mean edge lengths of 0.09 cm at the interface and 0.33 cm along the lateral wall. The volume meshes were then constructed using tetrahedral elements while preserving the refined surface resolution. The solid mesh comprises 138,847 nodes and 699,932 elements, while the fluid mesh for the full 3D FSI simulation contains 142,620 nodes and 749,771 elements. Figure 5(a) shows the external pressure profile, which includes a zero-pressure phase, a gradual increase phase, and a constant-pressure phase. As presented in Fig. 5(b), the relative error in the outlet flow rate shows a peak of 6.1% at the ends of the phases, but gradually reduces to within 1%. Figure 5(c) shows the temporal variations in pressure at the labeled points, and Fig. 5(d) illustrates the spatial distributions of pressure, radial deformation, and stress magnitude along a path from one inlet to the outlet. Across all selected time points, the relative errors in radial deformation remain within 0.2%. For the stress magnitude, the relative error remains within 5% except at $t_1$, which corresponds to the zero-pressure phase. Additional cross-sectional views of the displacement and stress magnitude fields for the patient-specific geometry are provided in Supplementary Fig. S5. For pressure, the relative error is observed to increase with distance from the outlet. However, the pressure results exhibit temporal trends similar to the full 3D FSI results. At the inlet where the error accumulates, excluding the transition periods, the relative error remains within the range of -11% to -13% and an increase in error is observed at the bifurcation points. Supplementary Fig. S6 presents that approximately 38% of the total pressure offset originates from bifurcation regions.

For radial deformation, the relative errors remained within 0.2% across all selected time points. For stress magnitude, the relative error remained within 5% at the selected time points, except at $t_1$, which corresponds to a zero-pressure phase. Additional displacement and stress magnitude fields on cross-sectional views of the solid domain at the selected time points are provided in Supplementary Fig. S5. The full 3D FSI simulation took 21,735 s to complete. In contrast, the multi-fidelity model finished the analysis in 471 s, accounting for approximately 2.17% of the time needed for the full 3D FSI simulation.

### Full-Cycle Pulsatile Simulation of Intermittent Pneumatic Compression

**Fig. 6:**
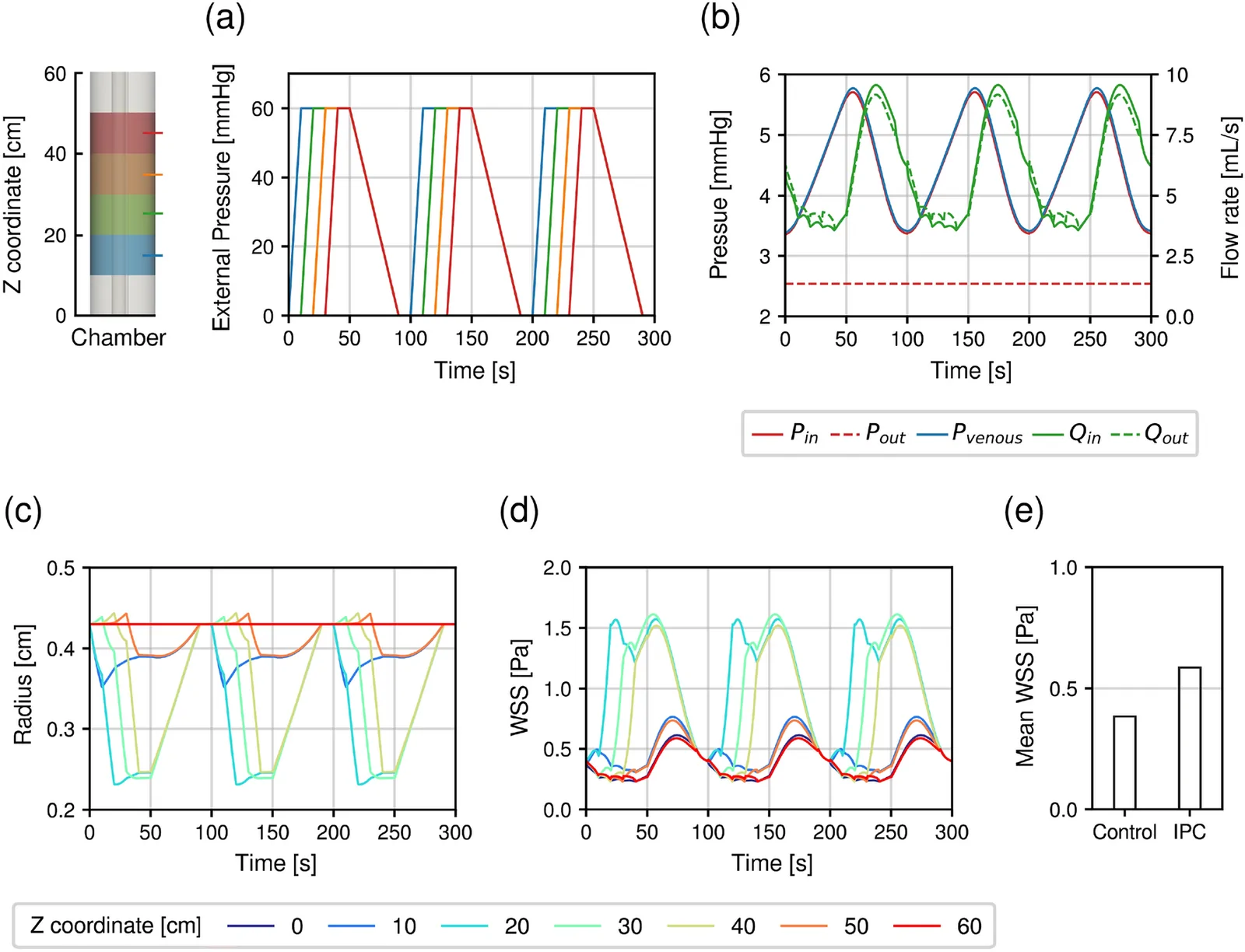
**a** External pressure applied to each chamber. The region of each chamber is indicated by color, and the corresponding external pressure is shown in the same color. **b**–**d** Results from a full-cycle pulsatile simulation of intermittent pneumatic compression on the simplified cylindrical model, illustrating (**b**) pressure and flow rate, (**c**) radius, and (**d**) wall shear stress (WSS) at specific points. **e** Comparison of WSS averaged over all centerline points and the full simulation duration, for the control case (left) and intermittent pneumatic compression (IPC) condition (right).

Full-cycle pulsatile simulations were performed using the simplified cylindrical model and subject-specific model to investigate tissue deformations and blood flow changes during IPC operation. The results of the simplified cylindrical model are first represented in Fig. 6. From 0 to 40 s, the radii of the veins decrease in regions where external pressure gradually increases, while adjacent regions exhibit a slight increase in diameter due to outward displacement of tissue, as shown in Fig. 6(c). As the radius decreases, the vascular resistance increases, leading to an increase in $P_{in}$. Consequently, the diminished pressure difference between $P_{venous}$ and $P_{in}$ results in a reduced flow rate. With decreasing cross-sectional area, $Q_{out}$ becomes higher than $Q_{in}$, as presented in Fig. 6(b). During 40 to 50 s, the radius of the vessel remains constant under maximum compression. In the previous phase, reduced $Q_{in}$ relative to $Q_{venous}$ resulted in blood accumulation in the LPN model, thus increasing $P_{venous}$. This in turn increased the pressure difference between $P_{venous}$ and $P_{in}$, leading to a corresponding increase in the flow rate. Between 50 and 90 s, the release of external compression results in a gradual increase in radius. This increase in the radius of the vessel reduces vascular resistance and consequently reduces $P_{in}$. As the pressure difference between $P_{venous}$ and $P_{in}$ increases further relative to the previous phase, the flow rate increases markedly, reaching 1.5 times $Q_{venous}$. With $Q_{in}$ exceeding $Q_{venous}$, a gradual decrease in $P_{venous}$ is observed. The wall shear stress (WSS) was estimated based on the Poiseuille flow assumption using the relation $\tau _w = 4\mu Q / \pi r^3$, where μ, *Q*, and *r* represent the dynamic viscosity, flow rate and radius, respectively. Figure 6(d) shows that the WSS is more affected by changes in the radius of the vessel than by flow rate. During the gradual increase phase, WSS increases in the compressed region despite a decrease in flow, whereas it decreases in the uncompressed region because of reduced flow. In contrast, during the decompression phase, the WSS declines in the compressed region as a result of an increase in radius despite increased flow, whereas it increases in the uncompressed region due to elevated flow. In addition, Fig. 6(e) illustrates that the WSS averaged across all centerline points and over time was 1.5-fold higher with the IPC device when compared to the control.

**Fig. 7:**
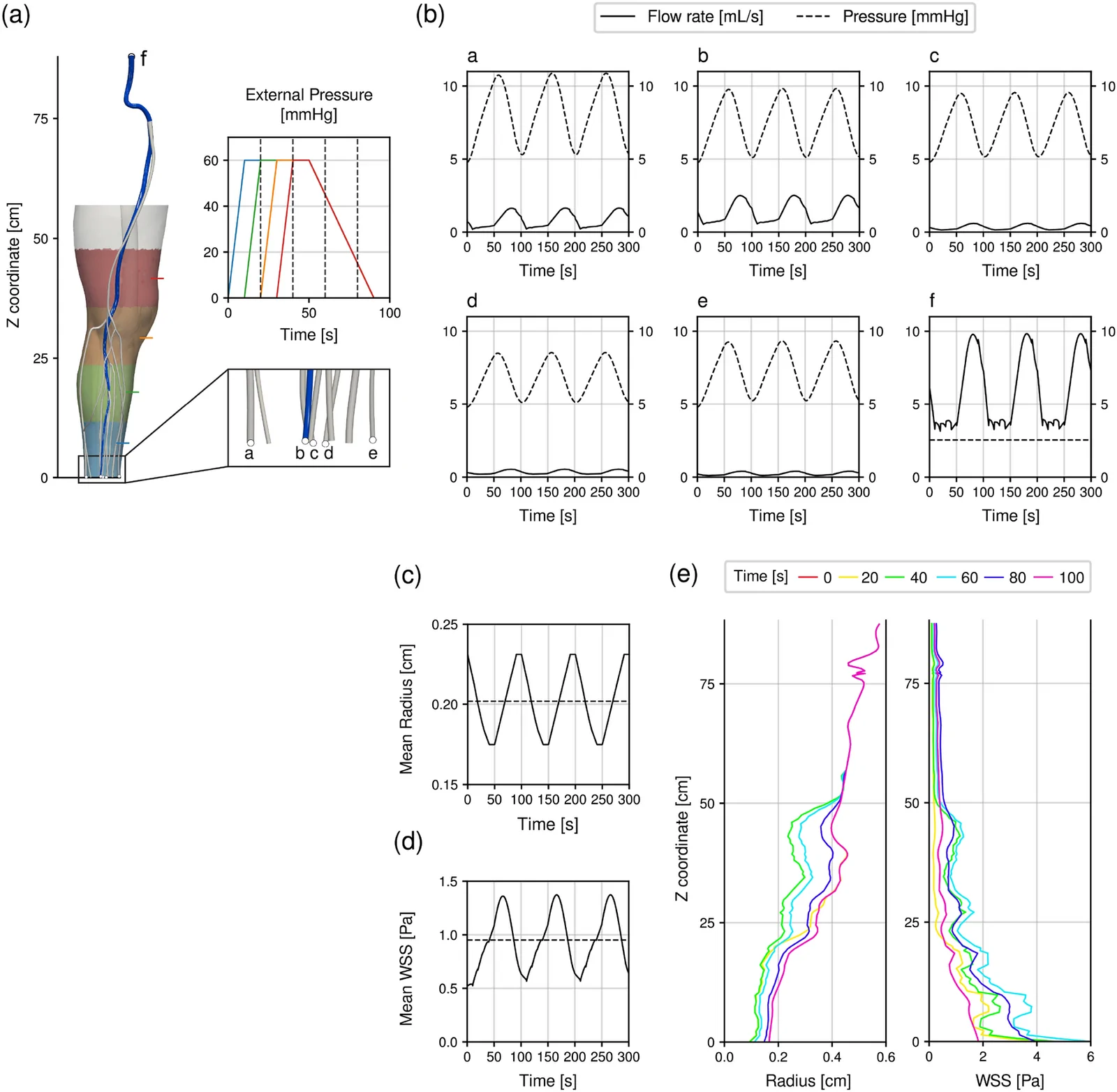
**a** External pressure applied to each chamber of the subject-specific model. **b** Calculated flow rate and pressure at the labeled inlets (**a**–**e**) and outlet (**f**). **c** The averaged radius and **d** wall shear stress (WSS) over all centerline points. The dashed lines indicate the time-averaged values. **e** Distributions of radius and WSS along the blue-marked path with respect to z-coordinate, from inlet (b) to the outlet, **f** at specific time points.

**Fig. 8:**
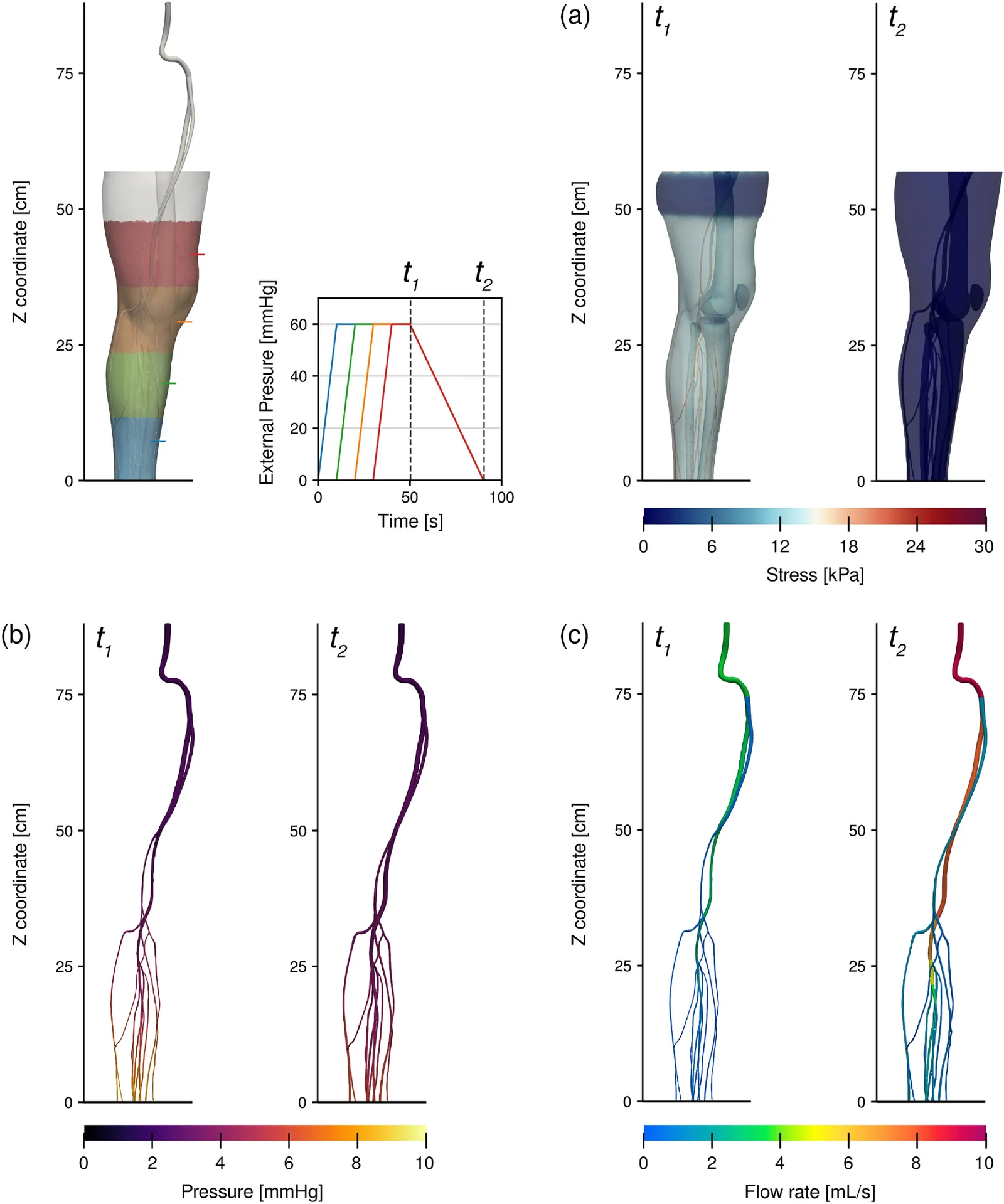
Simulation results of the subject-specific model, illustrating deformation and changes in **a** stress magnitude, **b** fluid pressure, and **c** flow rate at the end of the gradual increase phase ($t_1$) and the gradual decrease phase ($t_2$).

The results of the subject-specific model exhibit a pattern in flow rate and pressure similar to that observed in the simplified cylindrical model, as shown in Fig. 7(b). The outlet flow increased by up to approximately two-thirds. The pressure and flow rate at all the inlets are provided in Supplementary Fig. S7. Figure 7(c) presents the averaged radius and WSS over all centerline points. Compared to the control, which corresponds to the initial value, the averaged WSS across all centerline points was consistently higher with the IPC device. Specifically, the temporal averaged WSS was 1.8 times higher. The distribution of radius and WSS along the blue-marked path from one of the inlets to the outlet is illustrated in Fig. 7(d). Lastly, Fig. 8 visualizes the deformation and changes in stress magnitude, fluid pressure, and flow rate at the end of the gradual increase phase (40 s) and the gradual decrease phase (90 s). The results at all time points are provided in Supplementary Animation.

## Discussion

### Accuracy and Efficiency of the Multi-fidelity Model

We developed the multi-fidelity model by coupling 1D blood flow equations with 3D Cauchy equations of motion and compared it with the full 3D FSI simulations. As shown in Fig. 4, for the simplified cylindrical model, both the flow rate and pressure were nearly identical to those of the full 3D FSI simulations. In the case of the subject-specific model, as illustrated in Fig. 5, the multi-fidelity model exhibited a faster response to external pressure compared to the full 3D FSI results. However, the flow rate converged to comparable values after a short transition period. For pressure, a relatively constant offset was observed between the multi-fidelity model and the full 3D FSI results. The current model does not sufficiently address the additional pressure drop associated with bifurcations and non-circular cross sections, which likely contributes to the observed offset. The computational time analysis revealed that the multi-fidelity FSI model achieved a 9-fold speedup for the simplified cylindrical model, and an even greater 46-fold speedup for the more complex, subject-specific model.

These results demonstrate that the multi-fidelity model enables remarkably efficient computation while maintaining accuracy compared to the full 3D FSI simulation. This efficiency allows for a greater number of simulations within the same computational time, thereby facilitating parameter studies and optimization tasks. Furthermore, the more pronounced speedup observed in complex 3D geometries suggests that this approach is particularly advantageous for patient-specific analyses, significantly enhancing their feasibility.

### Full-Cycle Pulsatile Simulation of Intermittent Pneumatic Compression

We applied the multi-fidelity model to full-cycle pulsatile simulations of intermittent pneumatic compression using a simplified cylindrical and subject-specific geometries. The simulation of a full cycle, from 0 to 100 s, took 457 s for the simplified cylindrical model and 42.2 min for the subject-specific model.

Previous computational studies on intermittent pneumatic compression (IPC) therapy had struggles in modeling full-cycle pulsatile simulations due to intensive computational cost of the FSI simulation, complex geometries, and large temporal scales. To address these challenges, we utilized the multi-fidelity model for the full-cycle analysis of IPC operation. Furthermore, a lumped-parameter model of the distal venous system was implemented as the inlet boundary condition to realistically capture venous flow, accounting for the interaction between returning venous flow and external compression.

The results of this analysis provide mechanistic insight into IPC through full-cycle pulsatile flow simulations. As shown in Fig. 6(b), flow rate decreases during the gradual compression phase and rises sharply during relaxation, driven by accumulated venous blood volume. This cyclic operation induces periodic variations in flow, which may be optimized on a subject-specific basis depending on the clinical objectives of compression therapy. While previous studies have largely emphasized increased velocity during compression, few have examined hemodynamics across the entire IPC cycle [41, 42]. By performing full-cycle pulsatile simulations within a computationally feasible time frame, we achieve a more comprehensive understanding of IPC function.

The proposed methodology enables efficient and physiologically realistic simulations of externally induced tissue compression, offering a practical framework for the development and optimization of IPC devices and other compression-based therapies. Its computational efficiency supports patient-specific simulations, enhancing translational potential for medical device applications. Optimizing the efficacy and safety of medical devices requires not only reliable simulations but also quantitative evaluation, predictive accuracy, and explicit consideration of inter-individual variability. By enabling such analyses within a feasible computational time frame, the present model establishes a foundation for personalized simulations that can guide individualized treatment strategies.

This study also demonstrates clinical feasibility through its application to subject-specific CT data. Nevertheless, future work should integrate large-scale subject-specific clinical data to better capture patient heterogeneity and refine model parameterization, thereby enhancing predictive accuracy and generalizability. In addition, coupling this framework with AI-based approaches may enable real-time prediction and optimization, ultimately advancing clinical utility and accelerating the development of personalized healthcare solutions.

### Limitations

This study has several limitations. First, the current model does not fully incorporate the additional pressure drops resulting from bifurcations, non-circular cross sections, and other geometric complexities. This led to relatively consistent difference in pressure compared to the FSI results. The pressure difference may in turn affect the accuracy of the computed deformation and WSS. Previously, to improve the accuracy of 1D fluid models, the coefficients were adjusted based on 3D simulation [43], or additional pressure loss terms were introduced into the governing equations [44]. Future work will employ these methods to more accurately represent pressure loss. Second, the accuracy of the proposed multi-fidelity model was assessed through comparison with full 3D FSI simulations. Although previous studies have shown that FSI simulations of deformation and flow alterations under external compression correspond well with experimental and clinical observations [45, 46], the specific full-cycle compression configuration used in this study has not been experimentally validated. Moreover, the direct comparison with the full 3D FSI model was limited to the simplified benchmark problems and was not extended to the IPC simulation. Third, in the IPC simulation, we did not account for the flow changes caused by tissue deformation and the arterial and capillary network. In this study, $Q_{venous}$ was set to be constant in the LPN model of the distal venous system, based on the assumption that arterial flows are not significantly affected by the applied external pressure. However, accumulated venous blood volume and microvascular deformation may increase flow resistance and reduce the arteriovenous pressure gradient, which could in turn decrease $Q_{venous}$ and attenuate the associated increases in flow rate and WSS during the IPC cycle. In addition, external compression can influence capillary exchange by altering interstitial fluid pressure. To address this issue, some studies have simulated fluid flow from tissue using a poroelastic model [16, 17]. In a follow-up study, we will integrate the arterial and capillary network into the fluid domain and incorporate capillary exchange for a more physiologically realistic IPC simulation. Fourth, although the actual compression on the outer surface of the limb within the chamber may be non-uniform, a uniform external pressure was assumed for analytical simplicity. Finally, although the linear elastic model provided computational efficiency for evaluating the coupling strategy, it cannot fully represent the nonlinear and anisotropic mechanical response of biological soft tissues. Extending the framework to include more realistic constitutive models will be an important focus of future work.

### Concluding Remarks

In this study, we present a multi-fidelity fluid–structure interaction (FSI) model that explicitly couples a one-dimensional (1D) deformable blood flow formulation with the three-dimensional (3D) Cauchy momentum equation to efficiently and accurately simulate tissue deformation and flow alterations under external pressure. The framework was compared with full 3D FSI simulations in both simplified cylindrical and subject-specific geometries, demonstrating good accuracy while substantially reducing computational cost. It was further applied to full-cycle pulsatile simulations of intermittent pneumatic compression (IPC), effectively capturing dynamic changes across extended temporal scales. This approach enables efficient investigation of a wide range of biomechanical phenomena involving externally induced deformation and flow modulation, thereby improving mechanistic understanding of the underlying physiology. Due to its high computational efficiency, the proposed framework demonstrates clinical feasibility and provides a foundation for quantitative evaluation, predictive analysis, and explicit consideration of inter-subject variability in compression therapy and other biomechanics applications.

## Supplementary Information

Below is the link to the electronic supplementary material.Supplementary file 1 (mp4 1533 KB)Supplementary file1 (DOCX 5277 KB)

## Data Availability

Data supporting the results of this study are available from the corresponding author upon reasonable request. Materials supporting the results of this study are available from the corresponding author upon reasonable request.

## Code Availability

Source code developed in this study is available from the corresponding author upon reasonable request.
