# Supplementary material for: Efficient Multi-Fidelity Fluid–Structure Interaction Modeling for Pulsatile Blood Flow in Deformable Biological Tissues
Source: Ann Biomed Eng. 2026 Mar 11;54(9):3124–39. doi: 10.1007/s10439-025-03966-x (PMC13521970; doi:10.1007/s10439-025-03966-x)
Supplement: Supplementary file 1 [file 10439_2025_3966_MOESM2_ESM.docx]

Grid independence study of the 1D model

To evaluate the effect of spatial discretization on the 1D fluid model, a grid-independence study was performed by varying the point spacing along the centerline. For the simplified cylindrical model without bone, the spacing ranged from 0.25 to 2.0 cm, while for the patient-specific geometry it was further refined to 0.10 cm. Using the solution obtained at the smallest spacing as the reference, we computed the relative L2 norms of the inlet pressure and outlet flow rate across all time steps.

As shown in Fig. S1, the relative L2 norms decreased rapidly with mesh refinement. At a spacing of 0.5 cm, the relative L2 norms of the inlet pressure were below ${10}^{-3}$ for the simplified cylindrical model and below ${10}^{-2}$ for the patient-specific model, while the relative L2 norms of the outlet flow rate were below $3\times{10}^{-4}$ in both cases. Based on these results, a point spacing of 0.5 cm was selected for all subsequent simulations, as it provided an effective balance between accuracy and computational cost.

**Fig. S1** **(a)** Relative L2 norms of the inlet pressure and outlet flow rate for the simplified cylindrical model without bone, using the 0.25 cm spacing result as the reference. **(b)** Relative L2 norms of all inlet pressures and the outlet flow rate for the patient-specific model, using the 0.10 cm spacing result as the reference.

Determination of the lumped-parameter network model parameters

The parameters of the lumped-parameter network (LPN) model were determined as follows. To determine $Q_{venous}$, an initial inlet pressure $P_{in}$ was applied uniformly at all inlets in the absence of external compression. The value of $P_{in}$ was iteratively adjusted until the total inflow reached 6 mL/s, corresponding to the estimated mean flow to one leg [1]. The resulting inlet flow rate at each branch under this condition was then defined as $Q_{venous}$. To determine the elastance, we assumed that 5.6% of total blood volume (5 L) is contained in one leg in supine position [1, 2], and that 50% of this volume resides in the venous circulation represented by the LPN models. The venous blood volume assigned to each LPN model, $V_{venous},$ was distributed in proportion to the previously calculated $Q_{venous}$.The elastance was then calculated as $E=\left( P_{in}+Q_{venous} R \right)/V_{venous}.$ The inductance $L$ and resistance $R$ were selected to have minimal influence on the overall flow characteristics.

**Fig. S2** Parameters of the lumped parameter network model for **(a)** the inlet of the simplified cylindrical geometry and **(b)** each inlet of the subject-specific geometry. Q_venous_ represents the inflow (mL/s) into the model. E is elastance (dynes/cm^5^) and V_init_ is the initial venous volume (mL). L and R denote inductance (dyne⋅s^2^/cm^5^) and resistance (dyne⋅s/cm^5^), respectively.

Mesh-independence study for the simplified cylindrical model

To assess the sensitivity of the 3D solution to grid size, simulations were performed with several mesh configurations for the simplified cylindrical model without bone. The solution obtained with the finest mesh (mean edge length of 0.51 cm) was used as the reference, and the relative L2 norms of inlet pressure and outlet flow rate were evaluated across all time steps. As shown in Fig. S3, the errors decreased with mesh refinement. The mesh configuration adopted in this study was selected to achieve an appropriate balance between computational cost and accuracy. In this configuration, the relative L2 norms were within ${10}^{-2}$ for inlet pressure and $6\times{10}^{-3}$ for outlet flow rate.

**Fig. S3** Relative L2 norms of inlet pressure and outlet flow rate evaluated against the finest mesh for different mean edge lengths. The asterisk denotes the mesh configuration used in the study.

Comparison of the multi-fidelity model with full 3D FSI

**Fig. S4** **(a)** External pressure profile applied to the simplified cylindrical model. **(b)** Displacement and stress magnitude fields on an yz-plane slice at the specified time points ($t_{1}$-$t_{5}$), along with the corresponding error distributions relative to the full 3D FSI simulation.

**Fig. S5** **(a)** External pressure applied to the patient-specific geometry. **(b)** Displacement and stress magnitude fields on an yz-plane slice at the specified time points ($t_{1}$-$t_{4}$) and on three xy-plane slices located at 1/4, 2/4, and 3/4 of the model height at time point $t_{4}$, along with the error distributions relative to the full 3D FSI simulation.

**Fig. S6 (a)** External pressure applied to the patient-specific geometry. **(b)** Pressure error along the blue-marked path at time $t^{*}$. Dashed lines denote bifurcation locations along the path. **(c)** Temporal variation in the relative contributions of bifurcation and non-bifurcation regions to the total pressure error.

**Fig. S7** Flow rate and pressure at all inlets (a–j) and at the outlet (f) during the full-cycle pulsatile intermittent pneumatic compression simulation using the patient-specific geometry.

References

1.	Sharma, R., and S. Sharma. Physiology, Blood Volume. In: StatPearls. Treasure Island (FL): StatPearls Publishing, 2025

2.	Karpeles, L. M., and R. L. Huff. Blood Volume of Representative Portions of the Musculoskeletal System in Man. *Circulation Research* 3:483–489, 1955. https://doi.org/10.1161/01.RES.3.5.483
